# Factors Associated with No Dental Treatment in Preschoolers with Toothache: A Cross-Sectional Study in Outpatient Public Emergency Services

**DOI:** 10.3390/ijerph110808058

**Published:** 2014-08-08

**Authors:** Geovanna C. M. Machado, Anelise Daher, Luciane R. Costa

**Affiliations:** 1 Division of Pediatric Dentistry, Faculdade de Odontologia, Federal University of Goias, 1 av., Setor Universitario, 74605-220 Goiania-Goias, Brazil; E-Mail: geovannacm@yahoo.com.br; 2 Health Sciences Graduate Program, Federal University of Goias, 74605-020 Goiania-Goias, Brazil; E-Mail: anelisedaher@terra.com.br

**Keywords:** dental pain, children, dental health services

## Abstract

Many parents rely on emergency services to deal with their children’s dental problems, mostly pain and infection associated with dental caries. This cross-sectional study analyzed the factors associated with not doing an oral procedure in preschoolers with toothache attending public dental emergency services. Data were obtained from the clinical files of preschoolers treated at all nine dental emergency centers in Goiania, Brazil, in 2011. Data were children’s age and sex, involved teeth, oral procedures, radiography request, medications prescribed and referrals. A total of 531 files of children under 6 years old with toothache out of 1,108 examined were selected. Children’s mean age was 4.1 (SD 1.0) years (range 1–5 years) and 51.6% were girls. No oral procedures were performed in 49.2% of cases; in the other 50.8%, most of the oral procedures reported were endodontic intervention and temporary restorations. Primary molars were involved in 48.4% of cases. With the exception of “sex”, the independent variables tested in the regression analysis significantly associated with non-performance of oral procedures: age (OR 0.7; 95% CI 0.5–0.8), radiography request (OR 3.8; 95% CI 1.7–8.2), medication prescribed (OR 7.5; 95% CI 4.9–11.5) and patient referred to another service (OR 5.7; 3.0–10.9). Many children with toothache received no oral procedure for pain relief.

## 1. Introduction

Despite advances in dentistry over the last few decades, a large part of the population put off seeking dental care until they experience pain and discomfort [1,2,3]. Among these patients, a significant number of parents rely on emergency services to deal with their children’s dental problems [4,5,6,7,8]. Emergency dental services can be based in hospitals [9,10] or outpatient clinics [5,8,11]. According to Brazilian policies, general dentists working in primary care services should manage emergency dental problems. However, preschool children are at a particular development stage that can require specialized knowledge and training in pediatric dentistry to be appropriately managed [12].

Toothache is a major sign that dental caries is a chronic public health problem [13]. Because of its social impact, toothache is an oral health indicator [5,14,15]. Pain and infection caused by dental caries complications are what most motivate parents to seek emergency care for their children [1,5,14,15,16]. Dental pain can be provoked by tactile, chemical and thermal stimuli, or can appear spontaneously in cases of severe inflammation of the dental pulp [17]. In cases of provoked pain, the treatment of choice is sealing the cavity with restorative materials [17] whilst for spontaneous pain a tooth should receive pulp therapy or extraction to control the local infection [18]. These procedures should be performed in a dental emergency consultation, for the relief of pain.

Toothache interferes with important aspects of children’s development such as feeding [19,20], learning and leisure activities [19]. In cases of untreated caries in primary teeth, pulp involvement can occur quickly, with results ranging from provoked or spontaneous pain to pulp necrosis with fistula to facial cellulitis in more severe cases [5]. These situations significantly affect children’s quality of life [6,21,22], causing them and their families strong emotional and physical stress [23]. In addition, the non-treatment of primary teeth can endanger the health of permanent teeth; this represents an economic burden on society due to the cost of complex dental treatment in the future [2].

There are few studies in the literature about toothache treatment for preschool children in the public dental service. A study which presented dentists with a fictitious clinical case of toothache in primary molars compared the treatment plans of general American and British dentists for these teeth. On the first visit, both groups would perform drainage and prescribe antibiotics. On later visits, however, the Americans would perform more tooth restorations while the British would make more referrals to specialists and perform more dental extractions [24]. An Australian study showed that the number of emergency visits by children increased between 2008 to 2010, probably due to an increase in the prevalence of dental caries in primary teeth in poor communities in that country. Seventy to eighty percent of all toothache treatment in this study included radiographs, restorations and extractions [25]. That study [25] did not investigate the correlates of toothache management in young children though.

Health policies commonly describe that general dental practitioners should usually provide dental care for children at a primary level. Thus, considering the impact of dental caries in the quality of life of preschool children and the fact that many of them are not properly assisted by general practitioners, this cross-sectional study explored the factors associated with not managing toothache in preschoolers at public dental emergency services in a large Brazilian city.

## 2. Methods

The study with a cross-sectional design was approved by the Research Ethics Board of the Federal University of Goias (Protocol 111/2011). All 1108 records for children under 6 years of age who were seen at the nine dental emergency referral outpatient centers of the Municipal Health Bureau, Goiania-Goias, Brazil, from January to December of 2011, were examined. Goiania, capital of the state of Goias, is a city of about 1.3 million inhabitants located in central-western Brazil. Although the National Health Care System is directed to all Brazilian permanent residents and foreigners, underserved population largely uses public services, while people from middle and high socioeconomic status pay private plans to have access to medical and dental care. At these emergency referral outpatient centers, dental service is provided on weekdays in a conventional dental office not equipped with dental radiography devices. Dentists acting as general practitioners work 15-hour shifts treating the dental emergencies of adults and children.

According to the latest national oral health survey in Brazil, 5-year-old children in Goiania had a decayed, missing and filled teeth (dmft) index of 1.96, ranging from 1.62 to 2.30 [26]. The dmft index is the oral health parameter preconized by World Health Organization (WHO) for dentition status’ assessment to identify children with primary dentition presenting dental caries (decayed teeth), missing teeth as a result of caries and filled teeth after dental rehabilitation because of caries. In the most recent Brazilian national survey [27], dental caries was the most significant contributor to this index (78.1%), which suggests poor access to regular dental care.

Information about the children’s age and sex, chief complaint, teeth involved, procedures carried out, radiography request, prescription of medicines and patient referral was collected from dental records and inserted in a specific data collection tool. Only records indicating toothache related to dental caries as the chief complaint were included for data analysis.

The involved teeth were categorized as anterior and posterior. Information about procedures was classified in mutually exclusive options: no oral procedure (this category included orientation, uncooperative child not allowing treatment, beside exclusively radiography requests, prescription of medications, and referrals), tooth extractions, temporary restorations, or pulp procedures (including coronal opening, abscess drainage, pulpotomy and pulpectomy). Children that received a dental procedure could also have a radiography request, a drug prescription (not reported, analgesics or anti-inflammatories used alone, antibiotics, antibiotics associated with analgesics, analgesics associated with anti-inflammatories) and/or be referred to another health facility; those data were also recorded.

The data from the forms were put through descriptive statistical analysis and bivariate and logistic regression using IBM SPSS Statistics 19.0 (IBM Corporation, Armonk, NY, USA). The outcome studied was “the performance of oral procedures” using a yes/no dichotomy for different categories of clinical procedure. The independent variables were age (in ranges), sex, radiography request, medication prescription and patient referral. To evaluate the relationship between the independent variables and the performance of local clinical procedures, an association test was carried out using Pearson’s chi-square. The five independent variables were analyzed using a backward conditional logistic regression model to identify the risk of not performing a local clinical procedure on preschoolers with toothache. The odds ratio (OR) and the respective 95% confidence interval for each variable were estimated.

## 3. Results 

**Figure 1:**
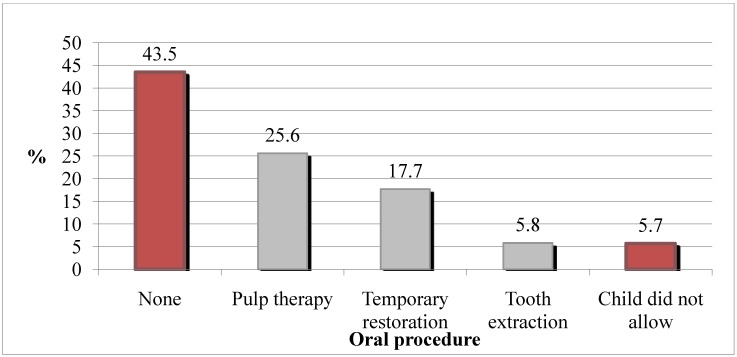
Frequency of procedures performed on preschoolers with toothache in community dental emergency services (nine cases excluded due to missing data).

Among the 1108 records examined, 531 stated toothache as the chief complaint (48.0%) and were included for further analysis. Preschool children attending emergency outpatient services because of toothache had a mean age of 4.1 (SD 1.0) years (minimum 1, maximum 5 years), and 274 were girls (51.6%). All teeth involved were primary: 54 (10.2%) were anterior and 257 (48.4%) were posterior; in 220 records the position of the tooth in the arch was not reported (41.4%). In 49.2% of the cases, no oral procedures were carried out, and in the other 50.8%, the most-reported interventions were pulp procedures and temporary restorations (Figure 1). Nine records (1.7%) did not report whether oral procedures were carried out; these nine cases were excluded from inferential analysis.

The remained 531 cases included 51 radiography requests (9.6%), 73 referrals (13.7%) and 215 cases in which medication was prescribed (40.5%). The type of medication prescribed was registered in 187 records (87.0%) and included analgesics or anti-inflammatories (31.6% of 215), analgesics and antibiotics (29.3%), antibiotics alone (20.0%) and analgesics associated with anti-inflammatories (6.0%). In 161 cases medication were prescribed but no oral procedure was carried out (Table 1).

Bivariate analysis showed that children who did not undergo oral procedures were younger than those who did. In addition, most dentists who did not carry out oral procedures requested radiographies, prescribed medication and gave the patient a referral, although there were dentists that performed oral procedures and also asked for radiographies, systemic medications and/or referred children to more specialized services (Table 2).

**Table 1:** Frequency of medication prescription in relation to the performance of oral procedures at community dental emergency services.

Prescription of Medication	Oral Procedure, n(%)		Total
	No	Yes	
No	96 (31.3%)	211 (68.7%)	307 (100%)
Yes	161 (74.8%)	54 (25.2%)	215 (100%)
Total	257	265	522 *

Note: * Of the 531 cases of toothache, 9 records provided no information about performance of oral procedures. For this reason, analysis was carried out with 522 cases.

**Table 2:** Analysis of the relationship between the independent variables and “performance of oral procedures”.

Independents Variables	Was There an Oral Procedure? n (% )		*p*-value
	No	Yes	
Age (categories)			0.023 *
1 to 2 years	22 (8.6%)	10 (3.8%)	
3 to 5 years	235 (91.4%)	255 (96.2%)	
Sex			0.140 *
Female	125 (48.6%)	146 (55.1%)	
Male	132 (51.4%)	119 (44.9%)	
Radiography requested	40 (15.7%)	11 (4.2%)	<0.001 *
Medication prescribed	161 (63.4%)	54 (20.5%)	<0.001 *
Patient referred to another service	57 (22.4%)	16 (6.1%)	<0.001 *

Note: * Pearson’s chi-square.

With the exception of “sex”, the independent variables tested in the regression analysis significantly associated with non-performance of oral procedures (Table 3): age (OR 0.7), radiography request (OR 3.8), medication prescribed (OR 7.5) and patient referred to another service (OR 5.7).

**Table 3:** Logistic regression model for non-performance of oral procedures on preschool children with toothache.

Variable	Odds Ratio	95% Confidence Interval for Odds Ratio		*p*-value
		Minimum	Maximum	
Male sex	0.7	0.5	1.1	0.087
Age (years, continuous variable)	0.7	0.5	0.8	<0.001
Radiography requested	3.8	1.7	8.2	0.001
Medication prescribed	7.5	4.9	11.5	<0.001
Patient referred to another service	5.7	3.0	0.9	<0.001

## 4. Discussion

In this study, slightly more than half the preschoolers received some oral procedure for their toothache, which suggests that the other group of children who did not have an intervention in their teeth came back home with a prescription or another recommendation instead of an immediate action to solve their problem. This is intriguing, because if they were seeking dental care for a painful tooth, they were supposed to receive a quick procedure (restoration, pulp therapy, extraction) [18] as soon as possible. This result agrees with a study in England, where just 60% of diagnosed children with dental pain received appropriate treatment [16]. This might indicate some sort of flaw in handling toothache emergencies, which is troubling given that studies report that emergency visits are still one of the main reasons for parents to take their children to the dentist [4,5,6,7,8]. It raises concerns because children from the present study may have suffered with toothache for a long time—another study reported that a child may have suffered with symptoms for more than two weeks when s/he is brought to a health service for relief of acute dental pain [10].

Currently, there is a discussion on the need to treat dental caries in primary teeth, because most of these teeth exfoliate normally without having been treated [28] or remain symptomless until shed naturally [29]. However, those studies [28,30] showed that untreated caries in young children can lead to toothache. In addition, there is evidence that children have a quick increase in their body weight and an improvement in their quality of life after cavities are managed [31]. A study in Scotland opposes the policy of non-intervention in primary teeth: it suggests that many cases of dental sepsis in 5-year-old children could be avoided by the treatment of dental caries in these teeth [32]. A Brazilian study showed that children’s dental care in public services is restricted to basic care procedures and the resolution of problems is low [33]. Those authors [34] also found that the children’s primary attention is focused on 6–12 years old, giving priority to permanent teeth. According to Duggal [34], “leave untreated disease as a matter of policy in young children, exposing them to the risk of pain, infections and a possible impact on their quality of life is just not an option”. 

Dentists in this study were less likely to perform oral procedures on younger children; they requested radiography, prescribed medication and/or referred the patient to another service instead. In the United States, many general dentists also do not treat very young children [35]. In that country, many children with toothache do not receive care due to cultural issues, lack of dental coverage or the unwillingness of health workers to treat them [36]. Since emergency services’ dentists are usually general practitioners, the fact that they do not treat younger children may be due to the difficulty that many dentists non specialists in pediatric dentistry have in dealing with possible behavioral problems. In very young children, anxiety during dental treatment and toothache are predictors of aversive behavior during dental treatment [37]. In the present study, less than 6% of dental charts cited that children did not allow dental treatment, but that could be the tip of the iceberg because dentists might have not written it down.

Emergency situations need to be dealt with quickly and many depend on a radiographic examination to complement the diagnosis. However, in the conditions of this study, radiographs would not be a priority if we consider that children were in pain secondarily to dental caries, teeth were primary, X-ray equipment was unavailable in the dental service, and the dentist could safely perform a temporary restoration or pulpotomy, or even a primary tooth extraction, without radiography. According to the present results, radiographic exams were requested in about one tenth of the cases of toothache in preschool children. This result differs from that of Sakai *et al.* [1], where the exam was performed in 53.6% of cases. In an Australian study that evaluated children’s emergency dental visits for 3 consecutive years (2008–2010), radiographic examination was also performed infrequently, in 18%, 21% and 28% of cases, respectively [25]. Blinkhorn and Zadeh-Kabir [24] showed that American general dental practitioners did radiographies in 98% of children’s emergency cases, while British dentists ordered dental radiographies in only 20% of visits. In those cases, dentists who did the most clinical interventions also did the most radiographic examinations [24].

The results of this study also demonstrate that dentists tend to limit themselves to prescribing medication in toothache emergencies, without performing any local intervention. Antibiotics, by themselves or in association with analgesics, were the most prescribed drugs for toothache according to emergency service records. These results are similar to those of Tulip and Palmer [16] who found that 98% of prescribed medications were antibiotics, either alone or in combination with analgesics. In an American study, toothache patients in emergency departments received palliative care in the form of analgesics and antibiotics [38]. That study also concluded that the prescription of these drugs had increased substantially over time [38]. In general clinical practice, lack of time and uncertainty about the correct diagnosis may be the main reasons for the prescription of antibiotics alone in cases of toothache [39].

Antibiotics should be administered as an adjunct to definitive treatment as they only treat the symptoms of the infections caused by caries [30]. In cases of inflammation of the dental pulp, an oral procedure is fundamental to relieve pain and it is not appropriate to prescribe only systemic medication [40], especially not antibiotics alone [41,42]. Nagle *et al.* [41] concluded that the use of antibiotics in untreated pulpitis is not able to reduce pain or the need to administer analgesics and therefore should not be prescribed alone to treat this condition. A recent systematic review concluded that there is no evidence that antibiotics could help in pain relief in patients with untreated irreversible pulpitis [42]. In addition, the improper prescription of antibiotics and analgesics in emergency services can trigger allergic reactions or toxicity in addition to the possibility of promoting resistance or intolerance to antibiotics [40].

Although many children in this study did not receive immediate oral treatment for their emergency, referral to other facilities was reported only rarely. This finding differs from that of Klaassen *et al.* [43], who report that children are increasingly being referred to specialized dentists for treatment. Those authors [36] report that the main reasons for these referrals are fear- and anxiety-related behavior problems with uncooperative patients. Furthermore, lack of knowledge about behavioral control techniques also favors the referral of these children [43]. It is possible that the dentists in this study may have made referrals without recording them in the files, as many files were incomplete. Posterior primary teeth were those most frequently involved in episodes of caries-related pain. These data agree with those from a previous study involving children of similar ages and are of concern because of the importance of these teeth in mastication and in preserving space in the dental arch for the eruption of the permanent successors [8].

Pulp intervention was the most frequently performed procedure, as in the study of Sqhair *et al.* [8]. This contrasts with the results of other studies in which caries restoration [1,25] or extractions [16] were the most common interventions. 

The major limitation of this study is the high possibility of loss of information due to incompletely filled out clinical records. Whereas we could include an acceptable sample size and did a systematic data collection, there is a chance that some of the variables analyzed were not properly registered. So, even though the results reported herein make sense and are supported by the literature, they should be viewed with caution. Besides, there is also the chance that dentists would have opted for an alternative procedure while the kids were heading to a more appropriate care. Also, this study design does not allow us to state that the child did not receive a proper dental intervention later on. Future longitudinal studies seeking causal factors for not performing an oral procedure in an emergency consultation, as well as following children after that first “palliative” care, might add to the present results.

Nevertheless, the results of this study should stimulate further work to evaluate the organization and planning of public dental services, contributing to the improvement of dental care for preschoolers with toothache. Maybe one of the key contributions of this study is that underserved preschoolers might have not received proper approaches for their toothache in a large city in Brazil, supporting recent results from other studies in other countries [2,4,9,16,34].

## 5. Conclusions

Under the conditions of this study, it was observed that many children received no immediate oral procedure to relieve toothache. The factors associated with dentists’ option for no dental treatment in preschoolers with toothache at public dental emergency services were: younger age, radiography request, prescription of medicines and patient referral to another service.
